# Author Correction: Breast cancer plasticity is restricted by a LATS1-NCOR1 repressive axis

**DOI:** 10.1038/s41467-023-35838-0

**Published:** 2023-01-10

**Authors:** Yael Aylon, Noa Furth, Giuseppe Mallel, Gilgi Friedlander, Nishanth Belugali Nataraj, Meng Dong, Ori Hassin, Rawan Zoabi, Benjamin Cohen, Vanessa Drendel, Tomer Meir Salame, Saptaparna Mukherjee, Nofar Harpaz, Randy Johnson, Walter E. Aulitzky, Yosef Yarden, Efrat Shema, Moshe Oren

**Affiliations:** 1 Department of Molecular Cell Biology, The Weizmann Institute of Science, 76100 Rehovot, Israel; 2 Department of Immunology and Regenerative Biology, The Weizmann Institute of Science, 76100 Rehovot, Israel; 3 Department of Life Sciences Core Facilities, The Nancy & Stephen Grand Israel National Center for Personalized Medicine (G-INCPM), The Weizmann Institute of Science, 76100 Rehovot, Israel; 4 Dr. Margarete Fischer-Bosch-Institute of Clinical Pharmacology and University of Tuebingen, Stuttgart, Germany; 5 Department of Immunology, The Weizmann Institute of Science, 76100 Rehovot, Israel; 6 Department of Pathology, Robert Bosch Hospital, Stuttgart, Germany; 7 Flow Cytometry Unit, Department of Life Sciences Core Facilities, The Weizmann Institute of Science, 76100 Rehovot, Israel; 8 Department of Cancer Biology, University of Texas MD Anderson Cancer Center, Houston, TX 77030 USA; 9 Department of Hematology, Oncology and Palliative Medicine, Robert Bosch Hospital, Stuttgart, Germany

**Keywords:** Tumour-suppressor proteins, Breast cancer, Tumour heterogeneity, Cancer models

Correction to: *Nature Communications* 10.1038/s41467-022-34863-9, published online 28 November 2022

In this article the acknowledgements of Efrat Shema are omitted and should have read ‘E.S is supported by the Emerson Collective and the Israel Cancer Research Fund, and is an incumbent of the Lisa and Jeffrey Aronin Family Career Development chair’. The original article has been corrected.

