# Supplementary material for: Breast cancer plasticity is restricted by a LATS1-NCOR1 repressive axis
Source: Nat Commun. 2022 Nov 28;13:7199. doi: 10.1038/s41467-022-34863-9 (PMC9705295; doi:10.1038/s41467-022-34863-9)

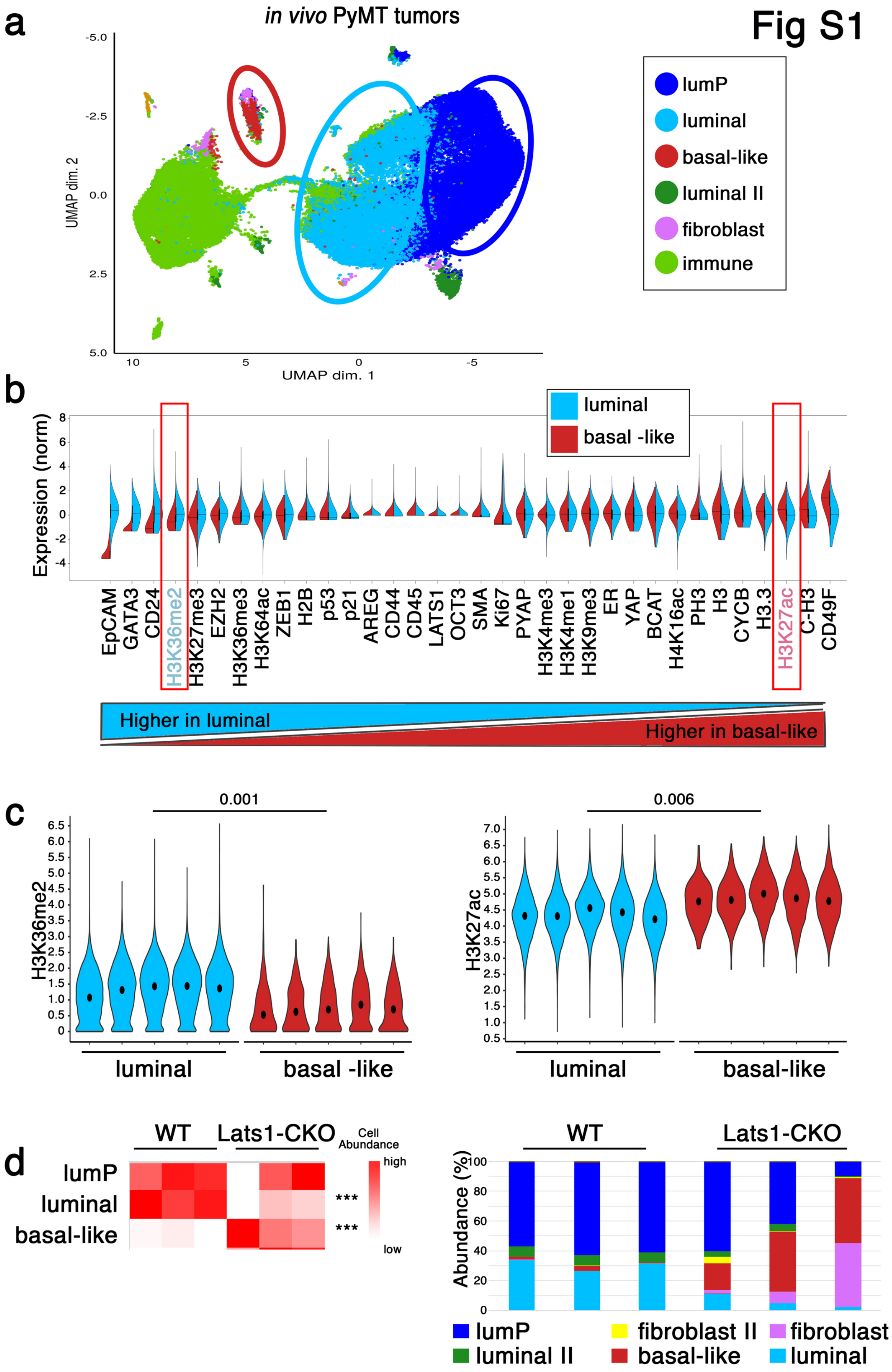
Fig S1
a
in vivo PyMT tumors
UMAP dim. 2
UMAP dim. 1
-5.0
-2.5
0.0
2.5
5.0
10
5
0
-5
lumP
luminal
basal-like
luminal II
fibroblast
immune
b
Expression (norm)
luminal
basal -like
EpCAM
GATA3
CD24
H3K36me2
H3K27me3
EZH2
H3K36me3
H3K64ac
ZEB1
H2B
p53
p21
AREG
CD44
CD45
LATS1
OCT3
SMA
Ki67
PYAP
H3K4me3
H3K4me1
H3K9me3
ER
YAP
BCAT
H4K16ac
PH3
H3
CYCB
H3.3
H3K27ac
C-H3
CD49F
Higher in luminal
Higher in basal-like
c
H3K36me2
0.001
luminal
basal -like
H3K27ac
0.006
luminal
basal-like
d
WT
Lats1-CKO
Cell Abundance
high
low
lumP
luminal
basal-like
***
***
Abundance (%)
WT
Lats1-CKO
lumP
luminal II
fibroblast II
basal-like
fibroblast
luminal

Figure S1: Epigenetic features of PyMT tumors. (a) Five WT PyMT tumors were dispersed into single cell suspensions and subjected to EpiTOF analysis as in Fig 1. (b) Luminal and basal-like subpopulations as defined in the EpiTOF analysis of *in vivo* tumor samples were compared. The expression of each of the markers was centered to have zero mean and is displayed in the form of a split violin plot ordered according to extent difference. Black lines designate medians. Red boxes denote H3K36me2 and H3K27ac. (c) Violin plot comparing mean expression levels of the indicated epigenetic marks in luminal vs basal-like *in vivo* EpiTOF subpopulations. Benjamini-Hochberg correction for multiple comparisons. (d) The relative abundance of cells in each subpopulation (within the total epithelial cell population) was compared in WT vs Lats1-CKO cells. Values were derived from EpiTOF data of WT and littermate matched Lats1-CKO tumor-derived cell lines and are presented as a heatmap (left panel), where darker red indicates a greater portion of the cells. Each column represents an independent cell line. Benjamini-Hochberg correction for multiple comparisons, *** p-value <0.01 within each subpopulation. In the right panel, the relative portion of each subpopulation is presented as a stacked bar graph, with each column representing an independent cell line of the indicated genotype. Source data are provided as a Source Data file.

Fig S2

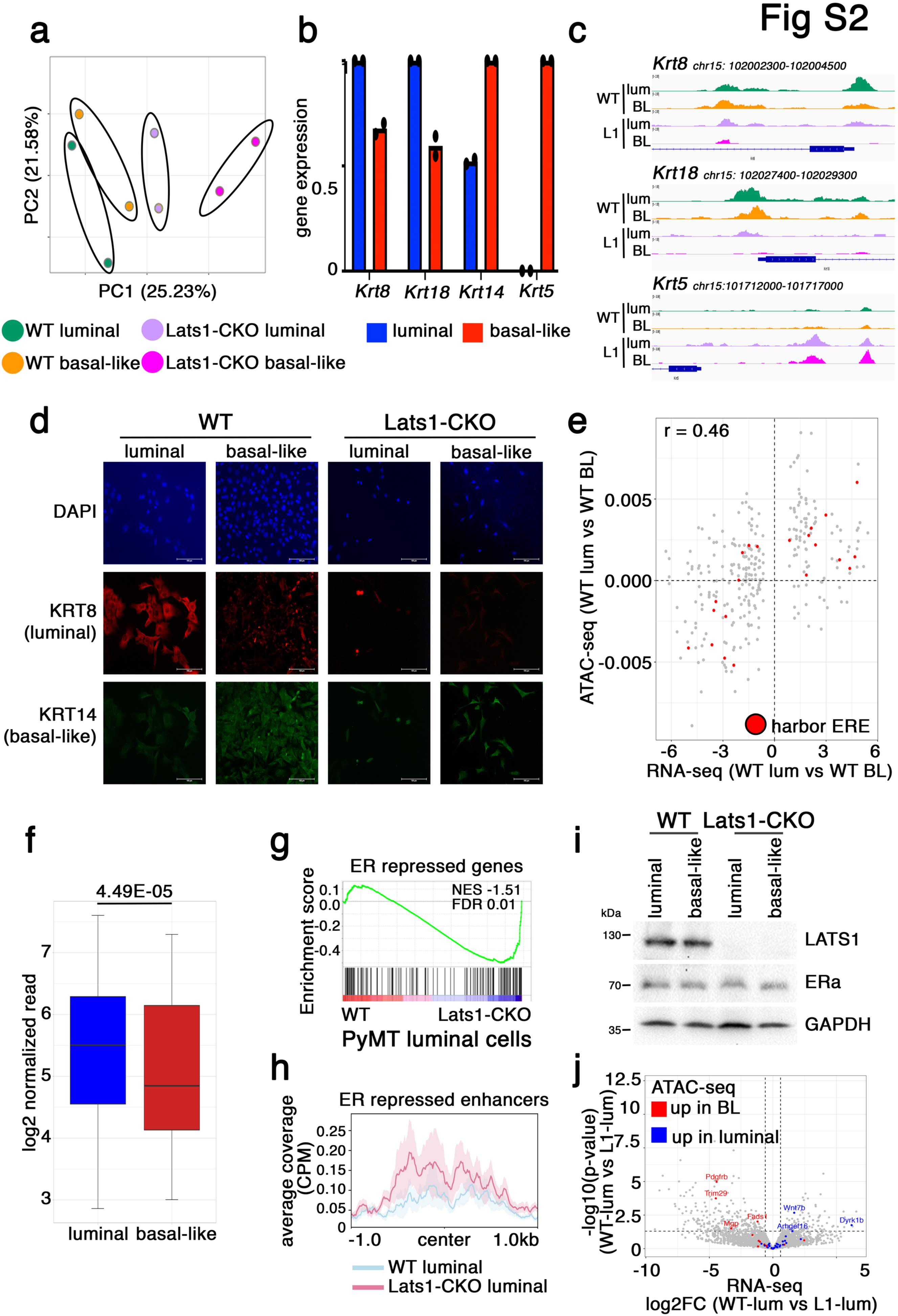

a
PC2 (21.58%)
PC1 (25.23%)
WT luminal
Lats1-CKO luminal
WT basal-like
Lats1-CKO basal-like
b
gene expression
0.5
0
Krt8
Krt18
Krt14
Krt5
luminal
basal-like
c
Krt8 chr15: 102002300-102004500
Krt18 chr15: 102027400-102029300
Krt5 chr15:101712000-101717000
WT
L1
lum
BL
d
WT
Lats1-CKO
luminal
basal-like
DAPI
KRT8 (luminal)
KRT14 (basal-like)
e
r = 0.46
ATAC-seq (WT lum vs WT BL)
0.005
0.000
-0.005
harbor ERE
-6
-3
0
3
6
RNA-seq (WT lum vs WT BL)
f
4.49E-05
log2 normalized read
7
6
5
4
3
luminal
basal-like
g
ER repressed genes
Enrichment score
NES -1.51
FDR 0.01
0.1
0.0
-0.2
-0.4
WT
Lats1-CKO
PyMT luminal cells
h
ER repressed enhancers
average coverage (CPM)
0.25
0.20
0.15
0.10
0.05
0
-1.0
center
1.0kb
WT luminal
Lats1-CKO luminal
i
WT
Lats1-CKO
luminal
basal-like
kDa
130
70
35
LATS1
ERa
GAPDH
j
ATAC-seq
up in BL
up in luminal
-log10(p-value) (WT-lum vs L1-lum)
12.5
10
7.5
5
2.5
0
Pdgfrb
Trim29
Fads1
Mgp
Wnt7b
Arhgef16
Dyrk1b
-10
-5
0
5
RNA-seq
log2FC (WT-lum vs L1-lum)

Figure S2: Characterization of luminal and basal-like subpopulations. (a) Two independent WT cell lines and Lats1-CKO cell lines each were used to generate luminal-enriched and basal-like-enriched cultures, using FACS sorting with EpCAM and CD49f antibodies. The enriched cultures were subjected to RNA-seq analysis. Principal Component Analysis (PCA) of global gene expression of the indicated cultures is presented. (b) RNA from WT luminal-enriched and WT basal-like-enriched cultures were subjected to RT-qPCR analysis of conventional luminal and basal-like genes. Values were normalized to *Hprt* ; *Krt8* and *Krt18* values in the luminal cultures and *Krt14* and *Krt5* values in the basal-like cultures, respectively, were set as 1.0. Mean ± SD of 2 biological repeats. Source Data file is provided. (c) Integrative Genomics Viewer (IGV) snapshots depicting ATAC-seq signals in the vicinity of the indicated genes in the four different cell subpopulations (WT = wild type PyMT, L1 = Lats1-CKO). For each gene, the Y-axis scale is identical for the different subpopulations. The associated RefSeq gene structure is presented below the tracks. (d) WT and Lats1-CKO enriched luminal or basal-like cultures were subjected to immunofluorescent staining. Scale bar = 100µm. Representative images of 5 biological repeats. (e) Genes differentially expressed in WT luminal (WT lum) compared to WT basal-like (WT BL) cells (RNA-seq analysis using *DESeq2,* raw p-value<0.05) were associated with ATAC-seq peaks present within 5kb of their TSS. Expression differences (log2 fold change) are plotted against accessibility differences between WT luminal and WT basal-like cells (log2 fold change), and peaks harboring an estrogen response element (ERE) are marked in red (HOMER[1] annotation). A total of 297 peaks are shown. (f) Genes contributing to the enrichment of the ERα activation signature in the GSEA analysis shown in figure 2e (left, 21 genes) were associated with ATAC peaks present within 5kb of their TSS (n= 40 peaks). For each peak, the mean read concentration (log2) in each condition was calculated. Boxplot shows median concentration of all peaks, lower and upper hinges correspond to the first and third quartiles and whiskers extend to the largest and smallest value. (g) Gene Set Enrichment Analysis (GSEA) of WT luminal vs Lats1-CKO luminal differential gene expression compared with Cicatiello et al “ER repressed” gene set[2]. (H) Cumulative ATAC-seq reads coverage of 72 enhancers of ER-repressed genes[2] in WT luminal, compared to Lats1-CKO luminal, cultures. Genes contributing to the leading edge of the GSEA in (g) were associated with enhancers using the ENC+EDP enhancer database[3]. Merged coverage from both replicates is shown. Lines depict average coverage, shaded areas represent SE. (i) WT and Lats1-CKO luminal-enriched and basal-like-enriched cultures were subjected to Western blot analysis with the indicated antibodies. GAPDH served as loading

control. Representative blot of 3 biological repeats. (j) Relative gene expression from RNA-seq in WT luminal vs Lats1-CKO luminal cultures was plotted according to magnitude of change (log2FoldChange, x-axis) and significance (*DESeq2,* -log10(raw p-value), y-axis). Dotted lines designate p-value < 0.05 and FC > 1.5, respectively. Genes associated with significantly differential ATAC-seq peaks (WT luminal vs WT basal-like cells as shown in Fig 2b (+/-5kb from TSS)) are colored according their increased accessibility in basal-like (BL) (red) or luminal (blue) cells.

Fig S3

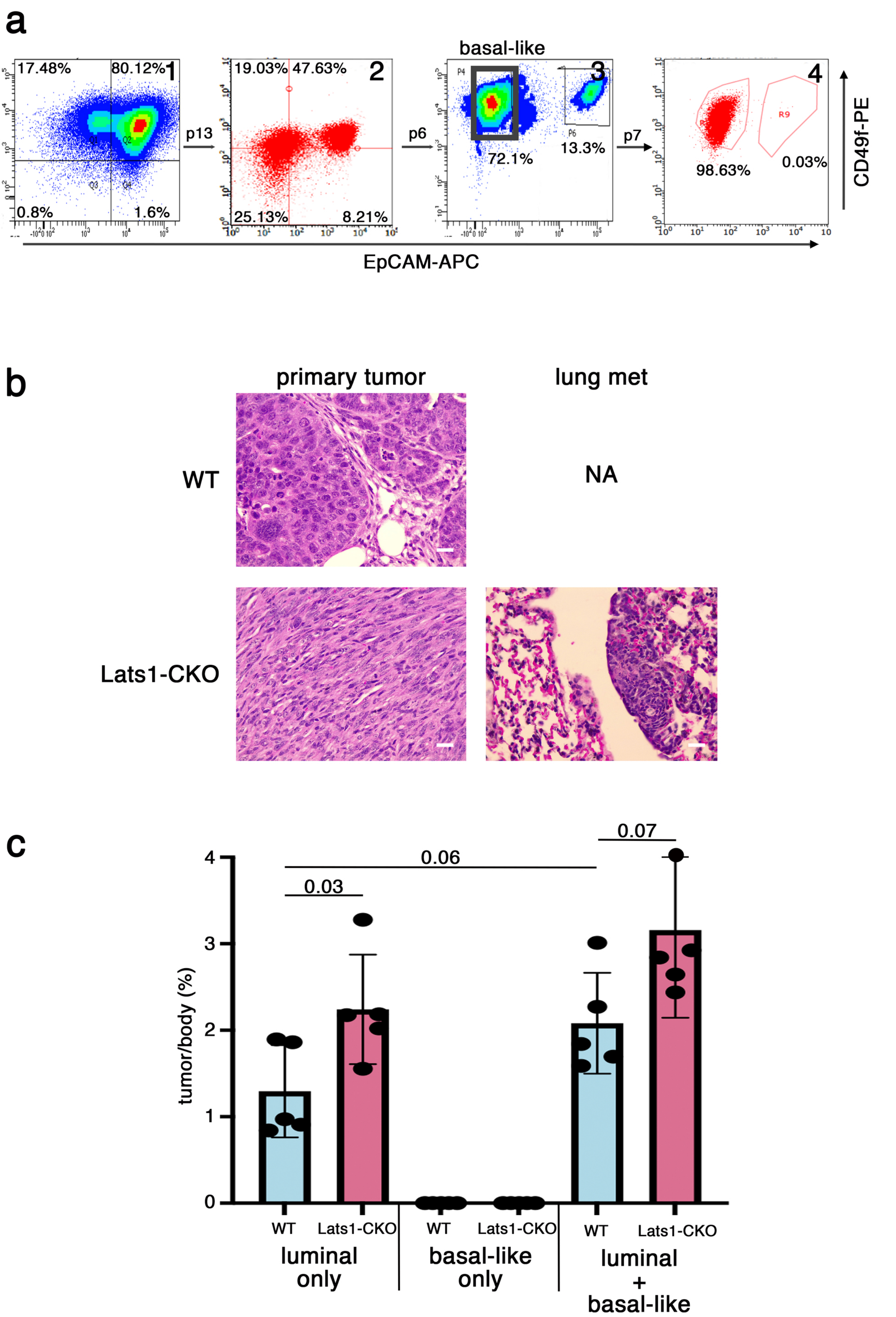
a
17.48%
80.12%
0.8%
1.6%
1
p13
19.03%
47.63%
25.13%
8.21%
2
p6
basal-like
72.1%
13.3%
3
p7
98.63%
0.03%
4
CD49f-PE
EpCAM-APC
b
primary tumor
lung met
WT
NA
Lats1-CKO
c
tumor/body (%)
0.07
0.06
0.03
WT
Lats1-CKO
luminal only
basal-like only
luminal + basal-like

Figure S3: Tumorigenic capacity of different types of PyMT tumor-derived cells. (a) Dissociated cells from a freshly harvested Lats1-CKO PyMT tumor were profiled by FACS (top, panel 1), using antibodies for EpCAM and CD49f. Basal-like cells were defined as $EpCAM^{low}CD49f^{high}$ (upper left quadrant). After a total of 19 passages in culture, the basal-like subpopulation was enriched by FACS sorting (panel 3), followed by 7 additional passages in culture and FACS analysis (panel 4). Bold black frame ("basal-like-sort") depicts the gates used for FACS-enrichment of the basal-like cell population. (b) WT or Lats1-CKO PyMT tumor-derived cell lines were injected into mammary fat pads of 5 female FBV/N mice. After 4 weeks, tumors were excised and examined histologically. Representative H&E stained sections of primary tumors or lung metastases are presented. "NA" = Not Applicable. Scale bar = 25µm (c) Luminal only, basal-like only or an equal number of luminal and basal-like cells of the indicated genotypes were injected into the mammary fat pads of female FBV/N mice. Four weeks post-injection, tumors were excised and weighed. Values represent average ± SE of 5 mice from each group. Two-way ANOVA was used to compute significance. Source data are provided as a Source Data file.

Fig S4

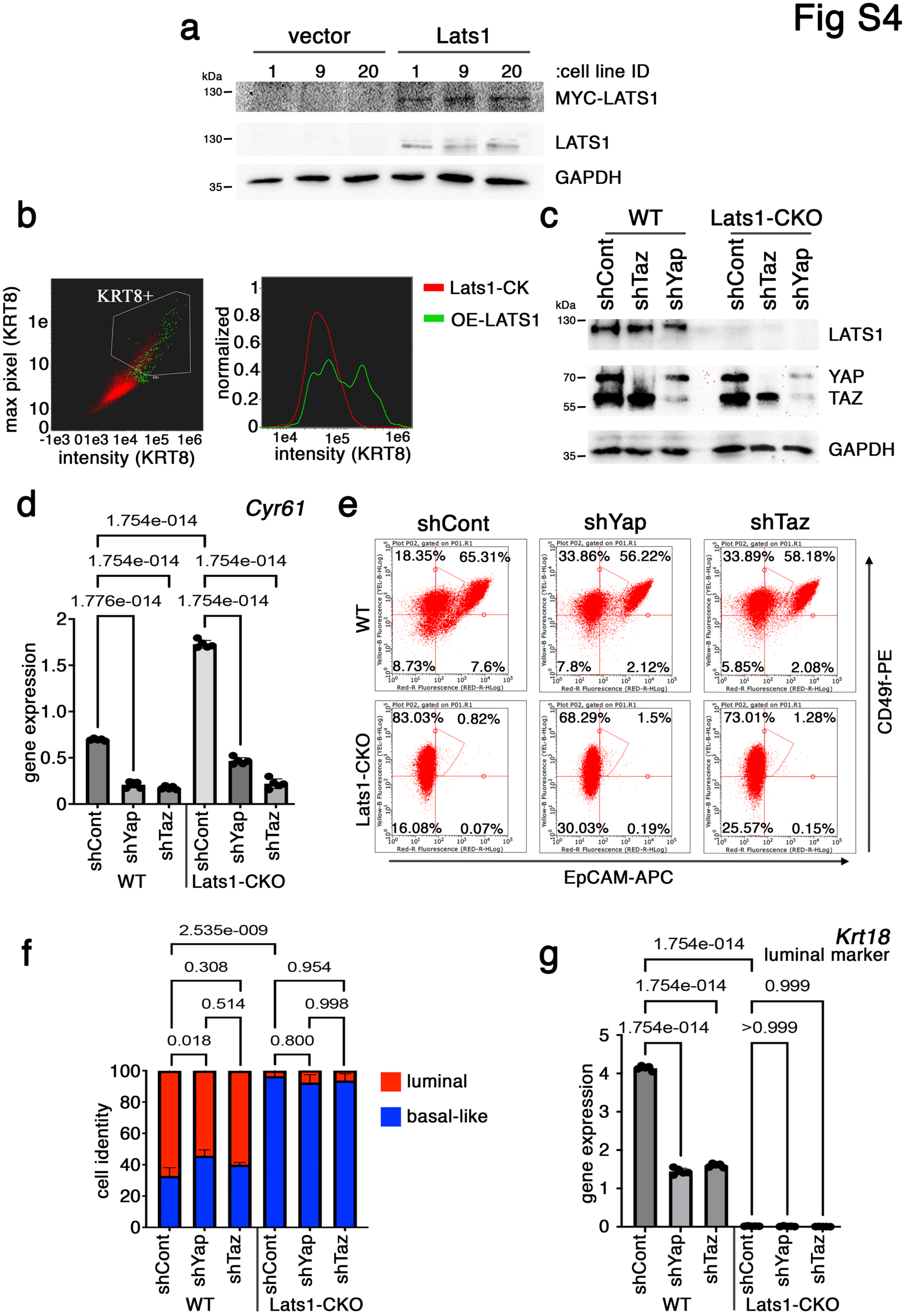

a
vector
Lats1
1
9
20
1
9
20
:cell line ID
kDa
130
MYC-LATS1
130
LATS1
35
GAPDH
b
KRT8+
max pixel (KRT8)
intensity (KRT8)
normalized
intensity (KRT8)
Lats1-CK
OE-LATS1
c
WT
Lats1-CKO
shCont
shTaz
shYap
shCont
shTaz
shYap
kDa
130
LATS1
70
55
YAP
TAZ
35
GAPDH
d
Cyr61
1.754e-014
1.754e-014
1.754e-014
1.776e-014
1.754e-014
gene expression
shCont
shYap
shTaz
shCont
shYap
shTaz
WT
Lats1-CKO
e
shCont
shYap
shTaz
WT
Lats1-CKO
18.35%
65.31%
8.73%
7.6%
33.86%
56.22%
7.8%
2.12%
33.89%
58.18%
5.85%
2.08%
83.03%
0.82%
16.08%
0.07%
68.29%
1.5%
30.03%
0.19%
73.01%
1.28%
25.57%
0.15%
CD49f-PE
EpCAM-APC
f
2.535e-009
0.308
0.954
0.514
0.998
0.018
0.800
cell identity
luminal
basal-like
shCont
shYap
shTaz
shCont
shYap
shTaz
WT
Lats1-CKO
g
Krt18
luminal marker
1.754e-014
1.754e-014
0.999
1.754e-014
>0.999
gene expression
shCont
shYap
shTaz
shCont
shYap
shTaz
WT
Lats1-CKO

Figure S4: LATS1 regulates phenotypic plasticity independently of YAP or TAZ. (a) MYC-tagged mouse *Lats1*, or vector control, was introduced into three independent Lats1-CKO tumor-derived cell lines. Levels of LATS1 were assessed by Western blot analysis using antibodies against LATS1 or MYC-tag (9E10). GAPDH served as loading control. Numbers above each lane denote parental cell line number. Representative blot of 2 technical repeats. (b) Lats1-CKO (red) PyMT cells were transiently transfected with MYC-tagged LATS1 (OE-LATS1, green). After 48 hours, cells were fixed and stained with anti-MYC and anti-KRT8 antibodies and subjected to imaging flow cytometry (ImageStreamX). Only single, focused cells with intact nuclei were analyzed. KRT8 positive (KRT8+) and OE-LATS1 (MYC+) cells were defined compared to an unstained control or a vector only control, respectively. Distribution pattern of cells expressing KRT8 (left; shape encompasses cells stained positively for KRT8) and intensity of KRT8 (right) staining was measured for each cell population. (c) WT or Lats1-CKO basal-like-enriched cells harboring control shRNA (shCont) or stable knockdown of *Yap* (shYap) or *Taz* (shTaz) were subjected to Western blot analysis with an antibody reactive with both YAP and TAZ. GAPDH served as loading control. Representative blot of 3 biological repeats. (d) Cells as in (c) were subjected to RT-qPCR analysis of expression of the YAP/TAZ target *Cyr61*. Values were normalized to *Hprt*. Average ± SE of 5 biological repeats. One-way ANOVA was used to calculate significance. Source data are provided as a Source Data file. (e) Cells as in (c) were analyzed by FACS, using EpCAM and CD49f antibodies. (f) Graphical representation of average relative numbers of luminal (upper right quadrant) or basal-like (upper left quadrant) cells, measured by FACS as in (e). Values represent average ± SD of 3 biological repeats. One-way ANOVA was used to calculate significance. Source data are provided as a Source Data file. (g) Cells as in (c) were subjected to RT-qPCR analysis of expression of the luminal marker *Krt18*. Values were normalized to *Hprt*. Average ± SE of 5 biological repeats. One-way ANOVA was used to calculate significance. Source data are provided as a Source Data file.

Fig S5

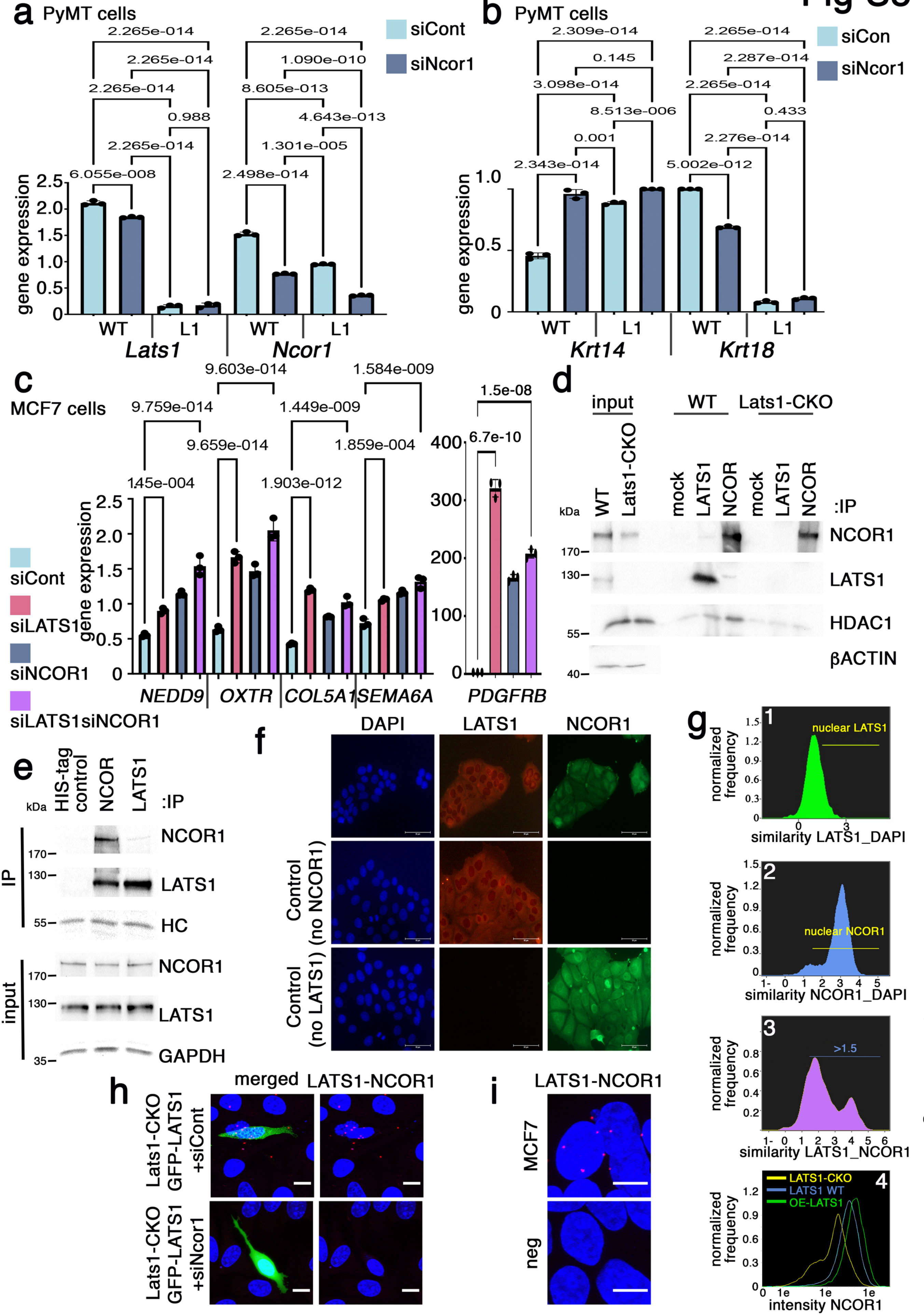

a PyMT cells
siCont
siNcor1
gene expression
Lats1
Ncor1
WT
L1
b PyMT cells
siCon
siNcor1
Krt14
Krt18
c MCF7 cells
siCont
siLATS1
siNCOR1
siLATS1siNCOR1
NEDD9
OXTR
COL5A1
SEMA6A
PDGFRB
d
input
WT
Lats1-CKO
mock
LATS1
NCOR
:IP
NCOR1
LATS1
HDAC1
βACTIN
kDa
e
HIS-tag control
NCOR
LATS1
:IP
IP
input
NCOR1
LATS1
HC
GAPDH
f
DAPI
LATS1
NCOR1
Control (no NCOR1)
Control (no LATS1)
g
normalized frequency
nuclear LATS1
similarity LATS1_DAPI
nuclear NCOR1
similarity NCOR1_DAPI
>1.5
similarity LATS1_NCOR1
LATS1-CKO
LATS1 WT
OE-LATS1
intensity NCOR1
h
merged
LATS1-NCOR1
Lats1-CKO GFP-LATS1 +siCont
Lats1-CKO GFP-LATS1 +siNcor1
i
LATS1-NCOR1
MCF7
neg

Figure S5: Transcriptional effects of knockdown of *Lats1* and *Ncor1*. (a) WT and Lats1-CKO PyMT cells were transfected with control siRNA (siCont) or siRNA against *Ncor1* (siNcor1). Three days later, an additional dose of siRNA was administered. Six days after the first transfection, cells were harvested for RT-qPCR analysis. Values were normalized to *Hprt*. WT siCont values were set as 1.0. Average ± SE of 3 biological repeats. One-way ANOVA was used to calculate significance. Source data are provided. (b) RT-qPCR analysis in the same cells as in (a). Average ± SE of 3 biological repeats. One-way ANOVA was used to calculate significance. Source data are provided. (c) MCF7 cells were transiently transfected with control siRNA (siCont) or siRNA against *LATS1*, *NCOR1* or both together. Two days later, cells were harvested for RT-qPCR analysis of LATS1-NCOR1-repressed genes. Values were normalized to *Hprt*. Average ± SE of 3 biological repeats. One-way ANOVA was used to calculate significance. Source data are provided. (d) Endogenous proteins were immunoprecipitated (IP) from WT or Lats1-CKO PyMT cells, followed by Western blot analysis with the indicated antibodies. "mock" denotes beads only (no antibody) with lysate. 2.5% of each lysate was run as "input". β-ACTIN served as loading control for input. Representative blot of 2 biological repeats. (e) Endogenous proteins were immunoprecipitated (IP) from MCF7 cells, followed by Western blot analysis with the indicated antibodies. A rabbit anti-HIS-tag antibody incubated with lysate served as a species-specific negative control. 2.5% of each lysate was run as "input". GAPDH served as loading control for input, and antibody heavy chain (HC) as a loading control for IP. Representative blot of 5 biological repeats. (f) MCF7 cells were subjected to immunofluorescent staining with antibodies against endogenous LATS1 (red) or endogenous NCOR1 (green) (top panels). Reactions lacking primary antibodies against NCOR1 (mid panels) or against LATS1 (lower panels) served as cross-reactivity controls. Representative images of 3 biological repeats. (g) Lats1-CKO PyMT cells were transiently transfected with MYC-tagged LATS1, and 48 hours later were stained with anti-MYC and anti-NCOR1 antibodies and subjected to imaging flow cytometry (ImageStreamX). Only single, focused cells with intact nuclei were analyzed. NCOR1 positive and MYC-LATS1 cells were defined compared to an unstained control or vector only controls, respectively. Similarity (correlation of staining pattern, see Materials and Methods) of LATS1 and NCOR1 with nuclear DAPI staining was used to assess cells with nuclear LATS1 (panel 1) or nuclear NCOR1 (panel 2). Similarity of staining patterns between LATS1 and NCOR1 was also measured (panel 3). The intensity of endogenous NCOR1 staining was measured in Lats1-CKO cells (panel 4, yellow), WT-PyMT cells (panel 4, blue) and Lats1-CKO cells

overexpressing MYC-LATS1 (OE-LATS1)(panel 4, green). Similarity >1.5 was considered significant. (h) Lats1-CKO cells were transiently transfected with GFP-tagged mouse *Lats1* together with control siRNA (siCont) or *Ncor1* siRNA (siNcor1). Colocalization of endogenous NCOR1 and GFP-tagged LATS1 was evaluated by Proximity Ligation Assay (PLA), using antibodies against NCOR1 and GFP. Scale bar = 10μm. Representative images of 2 biological repeats. (i) Colocalization of endogenous NCOR1 and LATS1 in MCF7 cells was evaluated by PLA. Reactions lacking an antibody against NCOR1 served as a negative control. Scale bar = 10μm. Representative images of 2 biological repeats.

Fig S6

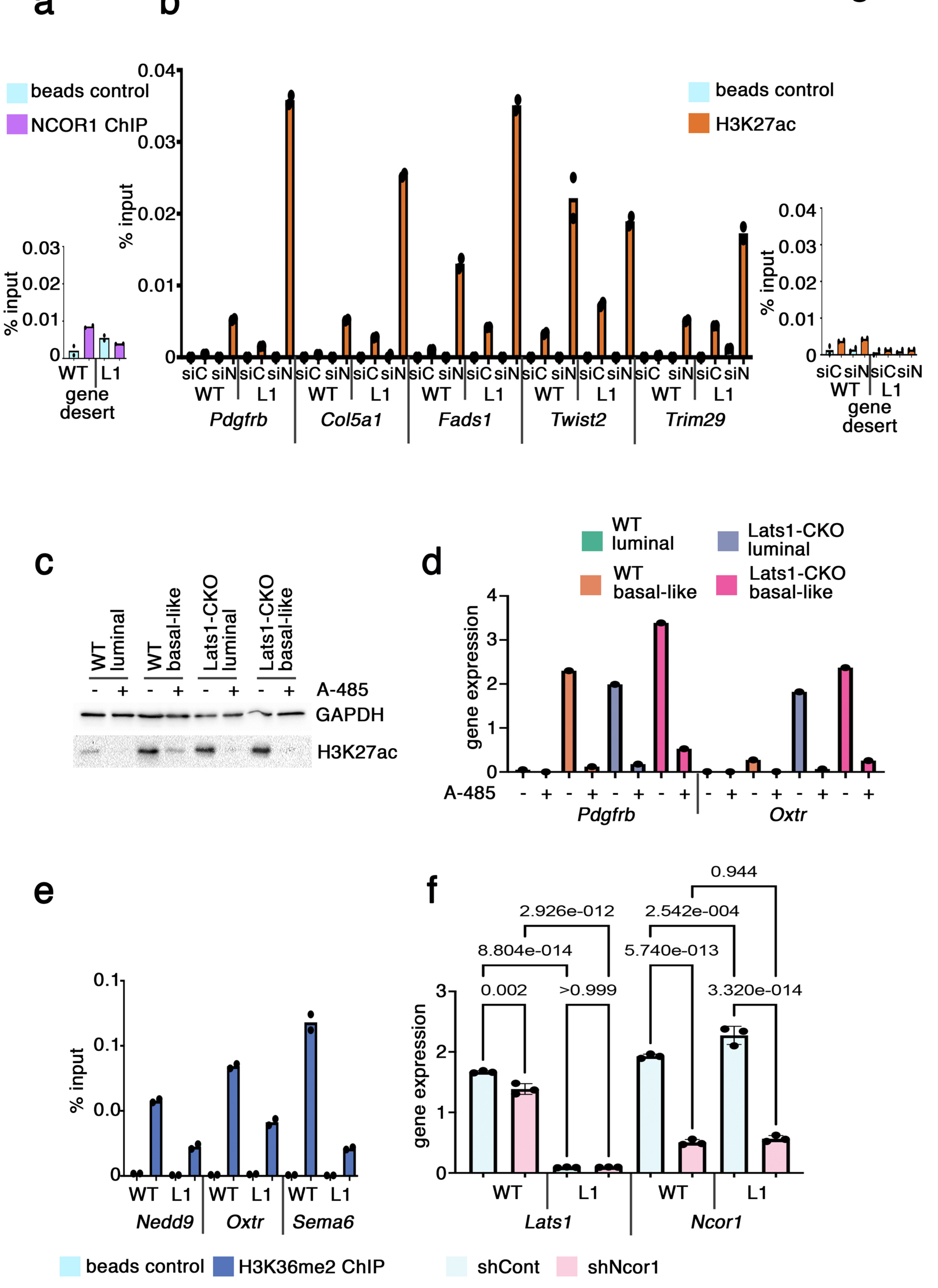
a
beads control
NCOR1 ChIP
% input
0.03
0.02
0.01
0
WT
L1
gene desert
b
0.04
0.03
0.02
0.01
0
% input
siC siN siC siN
WT L1
Pdgfrb
Col5a1
Fads1
Twist2
Trim29
beads control
H3K27ac
gene desert
c
WT luminal
WT basal-like
Lats1-CKO luminal
Lats1-CKO basal-like
A-485
- + - + - + - +
GAPDH
H3K27ac
d
WT luminal
WT basal-like
Lats1-CKO luminal
Lats1-CKO basal-like
gene expression
4
3
2
1
0
Pdgfrb
Oxtr
e
0.1
0.0
% input
WT L1 WT L1 WT L1
Nedd9
Oxtr
Sema6
beads control
H3K36me2 ChIP
f
0.944
2.926e-012
2.542e-004
8.804e-014
5.740e-013
0.002
>0.999
3.320e-014
gene expression
3
2
1
0
WT L1 WT L1
Lats1
Ncor1
shCont
shNcor1

Figure S6: ChIP and transcriptional analysis of ER-repressed genes. (a) WT and Lats1-CKO (L1) cells were subjected to chromatin immunoprecipitation (ChIP) with antibodies against NCOR1. qPCR of a gene desert region on chromosome 12, as well as beads without antibody, incubated with chromatin ("beads control"), were used as background controls. Values were normalized to input, and represent averages of 2 biological repeats. Source data are provided as a Source Data file. (b) WT or Lats1-CKO cells were transfected with control siRNA (siC) or siRNA against *Ncor1* (siN) for 48 hours, followed by ChIP with antibodies against H3K27ac. qPCR of indicated regulatory regions of specific genes (left panel) and of a gene desert region on chromosome 12, as well as beads without antibody, incubated with chromatin ("beads control"), were used as background controls (right panel). Values represent averages of 2 biological repeats. Source data are provided as a Source Data file. (c) WT and Lats1-CKO luminal and basal-like enriched cultures were treated with 3μM of the p300 inhibitor A-485. Cells were harvested after 24 hours and analyzed by Western blot for H3K27ac. GAPDH served as a loading control. Representative blot of 2 technical repeats. (d) The relative expression of LATS1-NCOR1-repressed genes was examined by RT-qPCR. *Gapdh* was used for normalization. Source data are provided as a Source Data file. (e) WT or Lats1-CKO cells were transfected as in (b) followed by ChIP with antibodies against H3K36me2. Controls and data analysis were as in (b). Values represent averages of 2 biological repeats. Source data are provided as a Source Data file. (f) WT and Lats1-CKO cells were infected with recombinant lentiviruses expressing control shRNA (shCont) or shRNA against *Ncor1* (shNcor1) and maintained under drug selection for at least 2 weeks, followed by RT-qPCR analysis of *Lats1* (left) and *Ncor1* (right) mRNA. Values were normalized to *Hprt* and represent the mean ± SE of 3 biological replicates. One-way ANOVA was used to calculate significance. Source data are provided as a Source Data file.

Fig S7

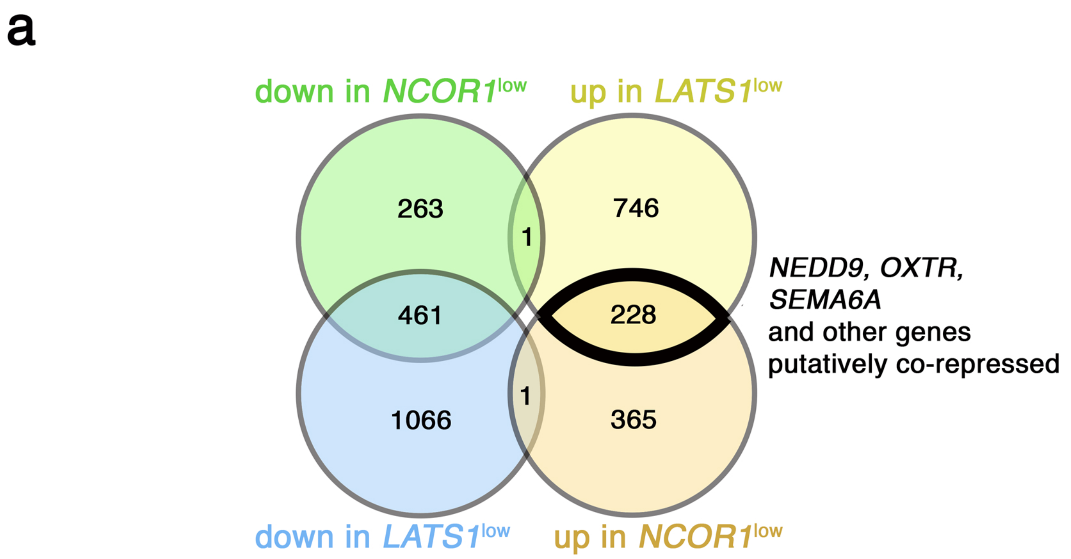
a
down in NCOR1low
up in LATS1low
263
1
746
461
228
1
1066
365
NEDD9, OXTR, SEMA6A
and other genes
putatively co-repressed
down in LATS1low
up in NCOR1low

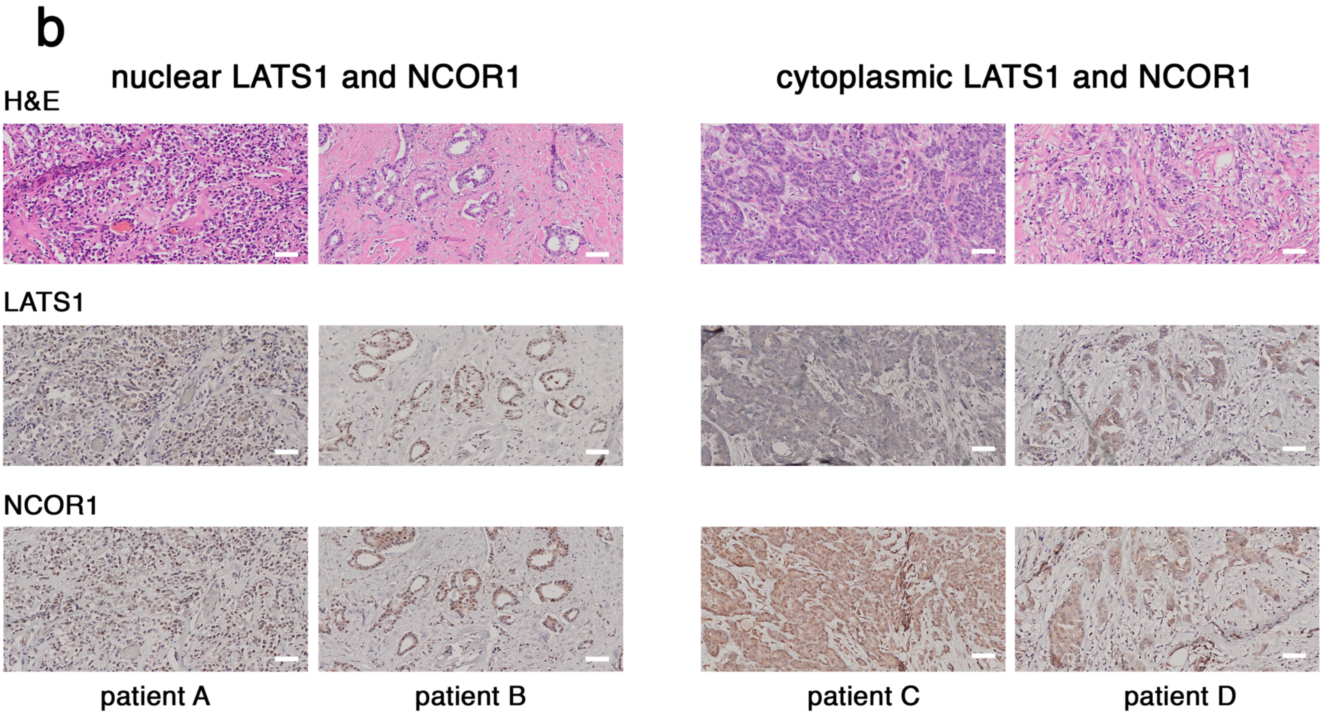
b
nuclear LATS1 and NCOR1
cytoplasmic LATS1 and NCOR1
H&E
LATS1
NCOR1
patient A
patient B
patient C
patient D

Figure S7: LATS1 and NCOR1 have similar effects on the expression of ER-repressed genes in human luminal breast cancer. (a) Comparison of gene expression in human luminal B tumors (BRCA-TCGA database) with low levels of *LATS1* ($LATS1^{low}$) or low levels of *NCOR1* ($NCOR1^{low}$). The Venn diagram depicts genes differentially expressed (FC>1.5, p-value<0.05). Top vs bottom quartiles of *NCOR1* or *LATS1* expression were used to define tumors with high or low *NCOR1* or *LATS1*, respectively. Numbers within regions represent number of genes. Bold black outline indicates the group of genes putatively co-repressed by LATS1 and NCOR1. (b) Immunohistochemistry (IHC) analysis of LATS1 and NCOR1 in human luminal breast tumors. Two representative tumor sections with nuclear LATS1 and NCOR1 (samples A and B) and two representative tumor sections with cytoplasmic LATS1 and NCOR1 (samples C and D) are presented (scale bar = 50µm).

References:

1 Heinz, S. *et al.* Simple combinations of lineage-determining transcription factors prime cis-regulatory elements required for macrophage and B cell identities. *Mol Cell* **38**, 576-589, doi:10.1016/j.molcel.2010.05.004 (2010).

2 Cicatiello, L. *et al.* Estrogen receptor alpha controls a gene network in luminal-like breast cancer cells comprising multiple transcription factors and microRNAs. *Am J Pathol* **176**, 2113-2130, doi:10.2353/ajpath.2010.090837 (2010).

3 Gorkin, D. U. *et al.* An atlas of dynamic chromatin landscapes in mouse fetal development. *Nature* **583**, 744-751, doi:10.1038/s41586-020-2093-3 (2020).

Table S1: Table of reagents used in study

Table S2: Number of reads and peaks, ATAC-seq

Table S3: ATAC-seq motif enrichment

Table S4: ATAC-seq ERE motif enrichments

Supplementary Table 1

EpiTOF antibodies

protein name	Name	Product	Provider	Metal
EpCAM	Anti- Mouse CD326 [EpCAM] (G8.8)-166Er	3166014	FLUIDIGM	166Er
CD49f	Anti-Human/Mouse CD49F (GoH3)-164Dy—100 Tests	3164006B	FLUIDIGM	164Dy
CD45	Anti-Mouse CD45 (30-F11)-89Y—100 Tests	3089005B	FLUIDIGM	89Y
CD24	Anti-Mouse CD24 150Nd (cancer stem cell marker)	3150009B	FLUIDIGM	150Nd
CD44	Anti-Human/ Mouse CD44 171Yb (cancer stem cell marker)	3171003	FLUIDIGM	171Yb
a-SMA	alpha SMA (Smooth muscle actin) (fibroblast marker)	ab5694	Abcam	173Yb
GATA3	Anti-Human/ Mouse GATA3 167Er	3167007A	FLUIDIGM	167Er
ERa	Anti-Estrogen Receptor alpha antibody [E115] - Low endotoxin, Azide free	ab167611	Abcam	146 Nd
beta-Catenin	Anti-Human/ Mouse/ Rat beta-Catenin 147Sm	3147005	FLUIDIGM	147Sm
CyclinB	Anti-Human/ Mouse CyclinB1	3153009A	FLUIDIGM	153eu
Ki-67	Anti- Human Ki-67 (B56) 162Dy	3162012	FLUIDIGM	162Dy
p53	Anti-Human p53	3143018	FLUIDIGM	143Nd
OCT3/4	Anti-Human/Mouse Oct3/4 (40/Oct-3)-165Ho 50 Tests	3165023A	FLUIDIGM	165Ho
AREG	Anti-Human/ Mouse AREG	LS-C341363	LSBio	159Tb
p21	Anti-p21 antibody [EPR18021] - BSA and Azide free	ab232512	Abcam	174 Yb
ZEB1	EMT azide and BSA free	NBP2-81015	Novus	158 Gd
YAP	D8H1X	CST-14074-BF	CST	161 Dy
EZH2	D2C9	CST-5246-BF	CST	144 Nd
LATS1	(C66B5)	CST-3477-BF	CST	169 Tm
pYAP (S127)	D9W2I	CST-13008	CST	151 Eu
H3	Anti-Histone 3 (D1H2)-176Yb 50 Tests	3176016A	FLUIDIGM	176Yb
pH3	Anti-Human/ Mouse/ Rat pHistone H3 [Ser28]	3175012A	FLUIDIGM	175Lu
cleaved H3	Rabbit monoclonal anti-cleaved-Histone H3 (Thr22) (clone D7J2K)	CST-12576 (custom f	CST	163Dy
H3K27ac	Acetyl-Histone H3 (Lys27) (D5E4) XP® Rabbit mAb #8173	CST-8173P	CST	160Gd
H3K36me3	Tri-Methyl-Histone H3 (Lys36) (D5A7) XP® Rabbit mAb #4909	CST-4909	CST	141Pr
H3.3	Rabbit monoclonal anti-Histone H3.3 (clone EPR17899)	ab176840	Abcam	155Gd
H3K4me3	Tri-Methyl-Histone H3 (Lys4) (C42D8) Rabbit mAb #9751	CST-9751	CST	145Nd
H3K27me3	Mouse monoclonal anti-trimethyl-Histone H3 (Lys27) (clone MABI 0323)	Active Motif-61017	Active Motif	168Er
H3K4me1	Mono-Methyl-Histone H3 (K4) (D1A9) XP(R) Rabbit mAb, 100 ul	CST-5326S	CST	154Sm
H3K9me3	Tri-Methyl-Histone H3 (Lys9) (D4W1U) Rabbit mAb #13969	CST-13969S	CST	170Er
H2B	Recombinant Anti-Histone H2B antibody [EP957Y] - BSA and Azide free	ab239842	Abcam	142Nd
H4K16Ac	Acetyl-Histone H4 (Lys16) (E2B8W) Rabbit mAb	CST-13534	CST	152Sm
H3K36me2	Di-Methyl-Histone H3 (Lys36) (C75H12) Rabbit mAb #2901	CST-2901	CST	149Sm
H3K64ac	Anti-Histone H3 (acetyl K64) antibody [EPR20713] - BSAand Azide free, 100 ug	ab251549	Abcam	156Gd

other antibodies

LATS1	(C66B5) Rabbit mAb	CST-3477	CST
LATS1	LATS1 antibody	HPA031804	Sigma
GAPDH	(14C10) Rabbit mAb	CST-2118	CST
MYC-tag	9E10	ab32	Abcam
H3	Anti-Histone H3 antibody - Nuclear Marker and ChIP Grade (ab1791)	ab1791	Abcam
H3K27ac	Acetyl-Histone H3 (Lys27) (D5E4) XP® Rabbit mAb	CST-8173P	CST
H3K36me2	Di-Methyl-Histone H3 (Lys36) (C75H12) Rabbit mAb (Alexa Fluor® 647 Conjuga	CST-15090	CST
ERa	Recombinant Anti-Estrogen Receptor alpha antibody [E115] - ChIP Grade (ab3206	ab32063	Abcam
EpCAM-APC	CD326 (EpCAM), mouse	130-102-234	Miltenyl
EpCAM-APC	anti human CD326 (Ep-CAM) 9C4	324208	Biolegend
CD49f-PE	human & mouse	130-119-767	Miltenyl
YAP/TAZ	YAP/TAZ (D24E4) Rabbit mAb	CST-8418	CST
GFP	Anti-GFP from mouse [9F9.F9]	ab1218	Abcam
NCOR1	NCoR1 Antibody	CST-5948	CST
NCOR1	NCoR1 Antibody	ab3482	Abcam
NCOR1	NCoR1 Antibody (F-1)	sc-515934	Santa Cruz
HDAC1	HDAC1 (D5C6U) XP® Rabbit mAb	CST-34589	CST
KRT8	Anti-Cytokeratin 8 Antibody, clone TROMA-1	MABT329	Sigma
KRT14	Anti-Cytokeratin 14 Antibody (LL001)	sc-53253	Santa Cruz
b-ACTIN	beta Actin Antibody monoclonal mouse nonconjugated	A00702	GenScript

qPCR primers

Cyr61	F-5' CGGAGGTGGAGTTAACGAGAAA	mouse, expression
	R-5' AAGACAGGAAGCCTCTTCAGTGAG	
Krt14	F-5' AGCGGCAAGAGTGAGATTTCT	mouse, expression
	R-5' CCTCCAGGTTATTCTCCAGGG	
Krt18	F-5' CAGCCAGCGTCTATGCAGG	mouse, expression
	R-5' CCTTCTCGGTCTGGATTCCAC	
Krt8	F-5' TCCATCAGGGTGACTCAGAAA	mouse, expression
	R-5' CCAGCTTCAAGGGGCTCAA	
Krt5	F-5' TCTGCCATCACCCCATCTGT	mouse, expression
	R-5' CCTCCGCCAGAACTGTAGGA	

Lats1	F-5' AGCAGCACGTAGAGAACGTCC	mouse, expression
	R-5' AATCCAACCCGCATCATTTC	
Ncor1	F-5' CTGCTCCGCATCAAGTGATAA	mouse, expression
	R-5' CCAGGAGTTCCCTGTGAGATA	
Ccna2	F-5' AAGAGAATGTCAACCCCGAAAAA	mouse, expression
	R-5' ACCCGTCGAGTCTTGAGCTT	
Oxtr	F-5' GGCCGTGTTCCAGGTTCTC	mouse, expression
	R-5' TGCAAGTATTTGACCAGACGAC	
Sema6	F-5' ACAGCCTGCCCCCTAAAGT	mouse, expression
	R-5' AGCTCCTCTTATATTCGAGCCC	
Pdgfrb	F-5' AGGAGTGATACCAGCTTTAGTCC	mouse, expression
	R-5'CCGAGCAGGTCAGAACAAAGG	
Ccna2	F-5' AAGAGAATGTCAACCCCGAAAAA	mouse, expression
	R-5' ACCCGTCGAGTCTTGAGCTT	
Fads1	F-5' AGCACATGCCATACAACCATC	mouse, expression
	R-5' TTTCCGCTGAACCACAAAATAGA	
Twist2	F-5' CGCTACAGCAAGAAATCGAGC	mouse, expression
	R-5' GCTGAGCTTGTCAGAGGGG	
Col5a1	F-5' CTTCGCCGCTACTCCTGTTC	mouse, expression
	R-5' CCCTGAGGGCAAATTGTGAAAA	
Nedd9	F-5' ATGTGGGCGAGGAATCTTATGG	mouse, expression
	R-5' TTCCCTGGGACAATGCCTTG	
NEDD9	F-5' ATGGCAAGGGCCTTATATGACA	human, expression
	R-5' TTCTGCTCTATGACGGTCAGG	
OXTR	F-5' CTGCTACGGCCTTATCAGCTT	human, expression
	R-5' CGCTCCACATCTGCACGAA	
SEMA6A	F-5' AATCAGTATTTCGCATGGCAACT	human, expression
	R-5' GCAATGTAGAGGGTTCCGTTCA	
COL5A1	F-5' GCCCGGATGTCGCTTACAG	human, expression
	R-5' AAATGCAGACGCAGGGTACAG	
PDGFRB	F-5' AGCACCTTCGTTCTGACCTG	human, expression
	R-5' TATTCTCCCGTGTCTAGCCCA	
Chr12 ChIP	F-5' CCATTGTTGGTGGGATTGC	gene desert
	R-5' TGAGGAACCGCCAGACTGAT	
Kisser ChIP	F-5' TCCCTCTCCGTAGACATGGG	TSS
	R-5' AAGGAGCCTTTCGCATCTCC	
Klh19 ChIP	F-5' TCCTTCCTTCTTCTCCCGGT	enhancer
	R-5' AACGACGACCACAGGTTGTT	
Oxtr ChIP	F-5' TGCTATGCCAAAGACCCCAG	promoter
	R-5' AGTGACAGCTTGGACGAAGG	
Sema6a ChIP	F-5' TACCAAAAAGGAACCCGGCA	enhancer
	R-5' TGTAAGTCCAAGCAGCGGAG	
Nedd9 ChIP	F-5' CCCTACTGTCCCCACTGCTA	enhancer
	R-5' GGAAACTCCACGTCACACCT	
Pdgfrb-TSS	F-5' TGGGGCAGGCCACTCTAATA	TSS
	R-5' GGACGCGTGTGTCTGTTTTC	
Col5a1-ChIP	F-5' AGCCAGAGCTGTTCAGATGTT	enhancer
	R-5' GAGCCTTCTGGAGCTCTCTTG	
Twist2-ChIP	F-5' AGACAAAACTGAAAGTGCCGC	TSS
	R-5' CCAGGGGCAGGACAAATTCT	
Trim29-ChIP	F-5' CAGTCTAGGCTGAAGCACCC	intron
	R-5' CCCAGGCCTGTCCCTAAATG	
Fads1-ChIP	F-5' TCATGGCAAGGACAGCGATT	TSS
	R-5' CGCTGGGGAACTCTTCACAT	

**plasmids**
pCMV-6myc-mouse LATS1 expression vector
pEGFP3C-full length MmLats1-WT
pcDNA III
pEGFP3C vector
pCDNA4TO-6xMyc-hsLATS1
pCDNA4/TO-Flag
pcDNA6/TR
pLP1 (packaging vector for lentivirus)
pLP2 (packaging vector for lentivirus)
pVSVG (packaging vector for lentivirus)
smart vector inducible non-targeting hEF1a turboRFP
Smart vector inducible shYAP
Smart vector inducible shTAZ (WWTR1)
PLKO1 shNCOR1 (mouse)

**knockdown sequences**

shYAP (seq GCATGAGACAGCTTCCATA)	
shNCOR1 (seq CGGCATAATCTTGACAACCTT)	
pLKO.1 eGFP shRNA control	
siCont	siRNA SMARTpool (Dharmacon)
siNcor1	siRNA SMARTpool siGENOME Mouse Ncor1 M-058556-01-0005 (Dharmacon)

Supplementary Table S2

Sample	Replicate	No. of reads (total)	Number of nucleosome-free reads (uniquely and properly paired)	No. of Peaks	
WT-lum	1	125,474,813	10,764,697	72,685	
WT-lum	2	101,645,671	7,514,288	58,783	
WT-Bas	1	125,581,278	16,786,894	70,446	
WT-Bas	2	124,679,765	6,606,816	51,497	
L1-KO-lum	1	111,389,522	2,159,884	14,753	* sample was excluded from further analysis
L1-KO-lum	2	157,106,727	8,967,914	93,467	
L1-KO-Bas	1	126,393,577	10,823,469	86,279	
L1-KO-Bas	2	125,802,680	6,137,454	99,022	

Supplementary Table 3
Motif enrichments Genomatix TF families
Z-Score >2 cuttoff

upLuminal	Z-Score (genome)	upBasal-like	Z-Score (genome)
ZF5F	20.75	AP1F	16.49
SP1F	19.65	NF1F	11.32
AP2F	19.09	ZF02	10.92
AP1F	18.08	HAML	10.7
NRF1	17.45	AP2F	10.64
ZF15	15.87	NOLF	10.22
AP1R	14.08	AP1R	10.01
BEDF	13.69	SP1F	9.99
EBOX	13.12	ZF11	9.41
ZF02	12.96	MYOD	9.19
EGRF	12.66	ZTRE	8.28
SNAI	12.58	CTCF	8.28
E2FF	12.4	KLFS	7.95
KLFS	12.34	EGRF	7.72
NF1F	12.12	ZF36	7.62
CTCF	10.85	AP4R	7.5
ZTRE	10.8	BEDF	7.48
XCPE	10.7	ZF29	7.4
ZICF	10.62	ZFXY	7.28
GLIF	10.52	HESF	6.88
ZFHX	9.92	ZF32	6.48
MAZF	9.7	MAZF	5.88
MYOD	9.7	NDPK	5.59
TF2B	9.65	ZF5F	5.5
MTEN	8.59	GLIF	5.36
NOLF	8.3	NFKB	5.36
BRAC	8.26	ZF64	5.27
GCF2	8.2	RBPF	5.23
XBBF	7.73	ZF07	5.18
HAND	7.55	GCF2	5.16
HESF	7.54	NEUR	5
HIFF	7.52	HAND	4.92
AHRR	7.38	SMAD	4.92
HDBP	7.2	P53F	4.85
ZF22	7.15	STAF	4.84
RXRF	7.03	MTEN	4.82
NDPK	7.01	ZF22	4.81
WHNF	6.93	EBOX	4.8
CDEF	6.82	MIZ1	4.78
NFKB	6.63	SREB	4.77
HASF	6.56	YBXF	4.56
ZF07	6.48	INSM	4.47
ZF43	6.42	PAX5	4.42

PAX9	6.39	ZICF	4.35
CALM	6.37	IKRS	4.31
SMAD	6.36	RP58	4.26
ZF29	6.17	CP2F	4.23
ZF36	6.01	NGRE	4.17
OAZF	6	ZF43	3.99
CHRE	5.85	HIFF	3.82
INSM	5.8	XCPE	3.6
E4FF	5.77	HICF	3.55
DEAF	5.7	E2FF	3.51
TALE	5.61	ZF19	3.48
AP4R	5.53	NACA	3.44
ZF64	5.52	KOX8	3.36
CP2F	5.47	ETSF	3.36
MIZ1	5.29	AHRR	3.28
MYRF	5.04	OAZF	3.26
VEZF	5.03	ZF42	3.24
FXRE	5.01	MZF1	3.14
SREB	4.97	FXRE	3.11
ZF11	4.96	SAL2	2.97
CREB	4.84	RXRF	2.97
NGRE	4.79	NRF1	2.94
PTF1	4.76	WHNF	2.89
ZF57	4.75	PAX9	2.85
RBP2	4.71	RBP2	2.77
ZFXY	4.61	PBXC	2.77
ZF32	4.35	MYRF	2.67
PEG3	4.33	TF3C	2.64
MEF3	4.29	ZF04	2.63
NEUR	4.01	NRSF	2.52
ZF37	3.97	ZF21	2.35
EREF	3.92	VEZF	2.28
ZF33	3.72	ZF08	2.18
BTBF	3.64	OSRF	2.16
ZBED	3.61	ZF20	2.07
HICF	3.5	NKRF	2.07
PAX5	3.46	TELO	2.06
MIRF	3.34	HUB1	2.01
CARE	3.24	MOKF	2
ESRR	3.2	PEG3	2
SAL2	3.09	PTF1	2
SF1F	2.97		
NACA	2.94		
ZF42	2.94		
PURA	2.92		
DICE	2.82		
TZAP	2.82		
NRSF	2.81		

PLAG	2.77
PERO	2.76
ETSF	2.67
PBXC	2.61
P53F	2.57
NR2F	2.48
RBPF	2.47
MITF	2.34
TELO	2.3
MTF1	2.29
DMTF	2.25
ZF47	2.23
PAX3	2.11
ZF04	2.08
ZF38	2.08
STAF	2.07
KOX8	2.06
TEAF	2.03

Supplementary Table 4

		UpLumPeaks		UpBasPeaks		
		424 matrices with z>2		363 matrices with z>2		
Genomatix name	motif	Over representation (genome)	Z-Score (genome)	Over representation (genome)	Z-Score (genome)	Comments/source
V$EREF	weighted from 5 motifs below	**1.53**	**3.92**	**1.28**	**1.83**	Weighted family score
V$ER.01	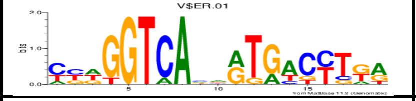	1.14	0.5	1.21	0.74	8 genomic binding sites
V$ER.02	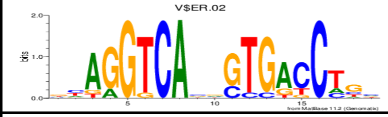	2.28	4.36	1.3	0.8	11 palindromic high affinity ERE binding sitesEstrogen gene activation
V$ER.03	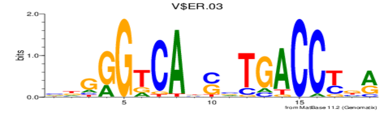	1.79	3.52	1.79	3.17	19 ChIP confirmed genomic binding sites; Most close to HOMER sequence
V$ER.04	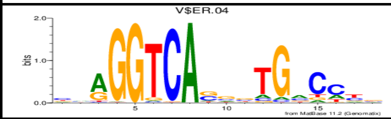	1.63	2.71	1.7	2.71	474 genomic binding sites identified by ChIP-seq approach
V$ESR2.01	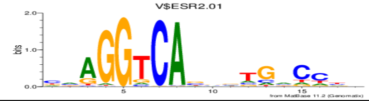	1.74	3.05	1.67	2.45	ER-beta targets; 4148 genomic binding sites identified by ChIP-seq

HOMER name	motif	Over representation (genome)	Z-Score (genome)	Over representation (genome)	Z-Score (genome)	Comments/source
ERE(NR) - HOMER	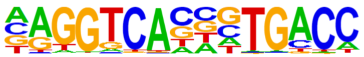	1.52	2.55	1.54	2.34	MCF7 ChIP

Supplementary Table 5
NCOR target list
Ingenuity Pathway Analysis (IPA, QIAGEN)
ATF3
AURKA
AXIN2
BCL6
BIRC3
BIRC5
BUB1B
BUB3
C3
CAV1
CCNA2
CCNB1
CCNB2
CCND1
CCNE2
CD44
CDCA3
CDH1
CDIPT
CDKN1A
CDKN2A
COL2A1
CXCL8
CYB5A
DBI
DHFR
DLGAP5
E2F1
ECT2
EGFR
EGR1
EHD1
EPB41L1
FOXM1
GADD45A
HES1
HMMR
IGFBP3
IGFBP3
IL1B
INSIG1
ISG15
ITGB4
JUN
JUP

KIF2C
KISSER
KLF6
LCN2
LGALS3BP
LGR5
MAPK8IP3
MDM2
ME1
MT1E
MT1X
NEDD9
OXTR
PBK
PLS3
PMAIP1
PPARG
PSIP1
RARA
RGS10
RRM2
RUNX2
S100A9
SAT1
SEMA6A
SERPINE1
SOX11
SOX9
SPTSSA
SWAP70
TOP2A
TTK
TYMS
UCP3
ZNF160

List of Western blots in **Breast cancer plasticity is restricted by a LATS1-NCOR1 repressive axis**

Fig S2D

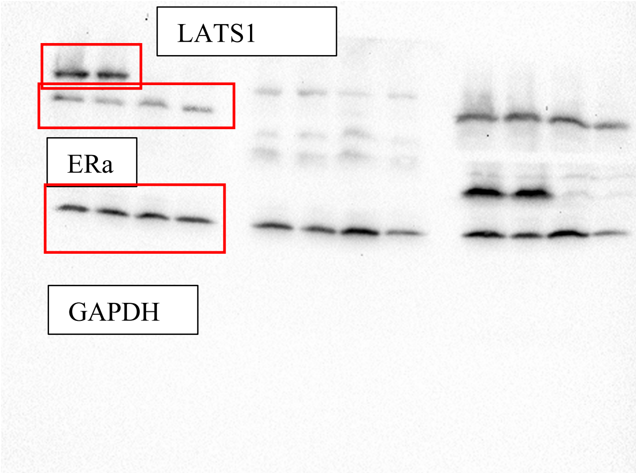

Fig S4A

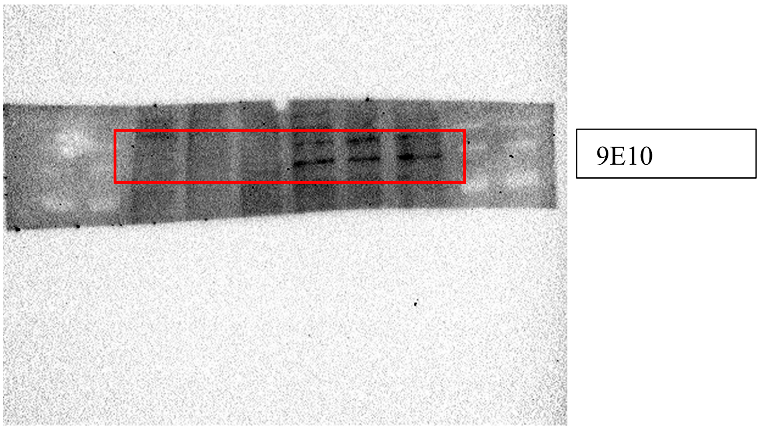

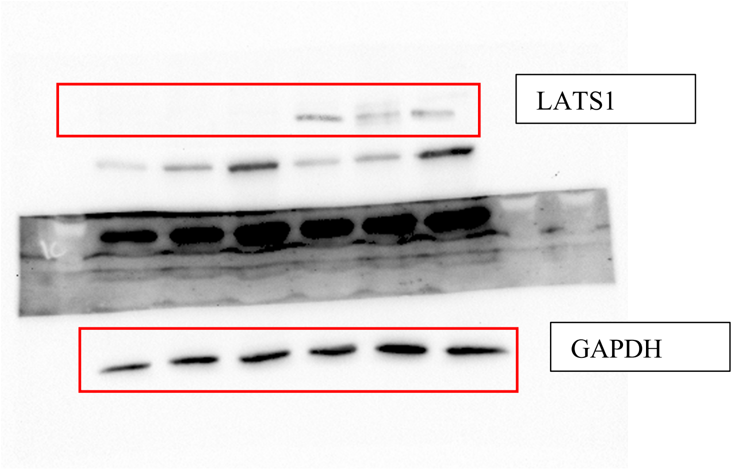

Fig S4B

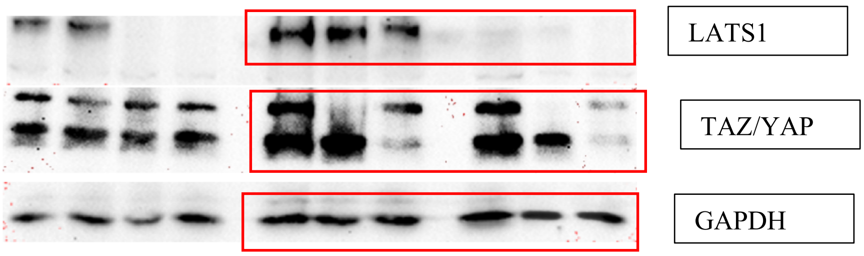

Fig S5D

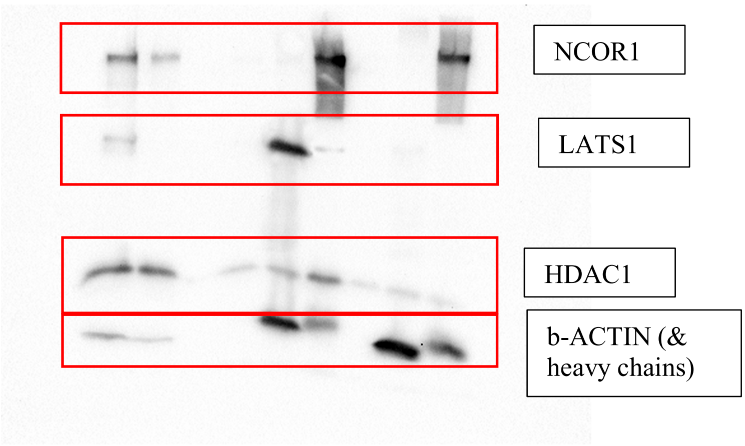

Fig S5E

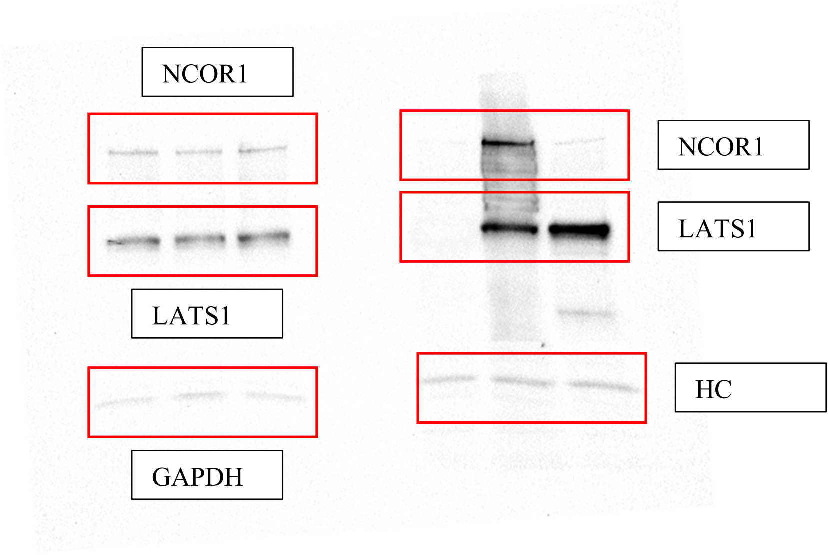

Fig S6C

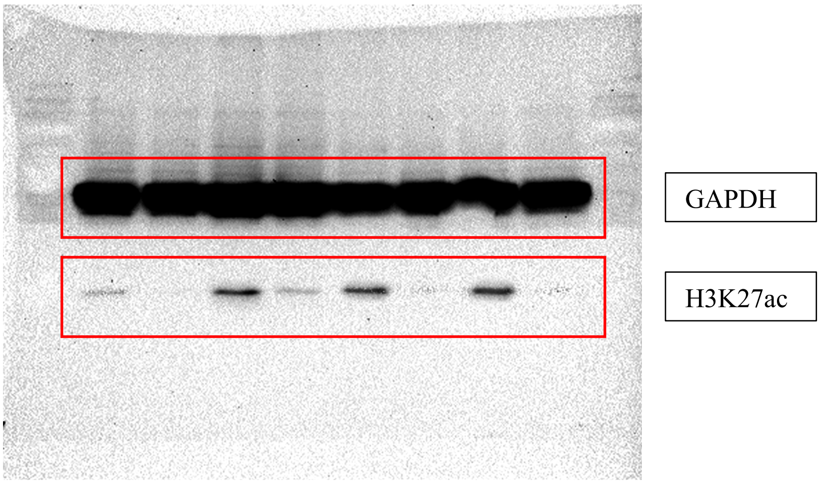

Supplement: Supplementary file 1 — Supplementary Information [file 41467_2022_34863_MOESM1_ESM.pdf]
