# Supplementary material for: Breast cancer plasticity is restricted by a LATS1-NCOR1 repressive axis
Source: Nat Commun. 2022 Nov 28;13:7199. doi: 10.1038/s41467-022-34863-9 (PMC9705295; doi:10.1038/s41467-022-34863-9)
Supplement: Supplementary file 3 — Reporting Summary [file 41467_2022_34863_MOESM3_ESM.pdf]

nature portfolio

Corresponding author(s): Yael Aylon and Moshe Oren

Last updated by author(s): 23 Oct, 2022

Reporting Summary

Nature Portfolio wishes to improve the reproducibility of the work that we publish. This form provides structure for consistency and transparency in reporting. For further information on Nature Portfolio policies, see our Editorial Policies and the Editorial Policy Checklist.

Statistics

For all statistical analyses, confirm that the following items are present in the figure legend, table legend, main text, or Methods section.

n/a	Confirmed	
☐	☒	The exact sample size (n) for each experimental group/condition, given as a discrete number and unit of measurement
☐	☒	A statement on whether measurements were taken from distinct samples or whether the same sample was measured repeatedly
☐	☒	The statistical test(s) used AND whether they are one- or two-sided<br>*Only common tests should be described solely by name; describe more complex techniques in the Methods section.*
☒	☐	A description of all covariates tested
☒	☐	A description of any assumptions or corrections, such as tests of normality and adjustment for multiple comparisons
☐	☒	A full description of the statistical parameters including central tendency (e.g. means) or other basic estimates (e.g. regression coefficient) AND variation (e.g. standard deviation) or associated estimates of uncertainty (e.g. confidence intervals)
☐	☒	For null hypothesis testing, the test statistic (e.g. F, t, r) with confidence intervals, effect sizes, degrees of freedom and P value noted<br>*Give P values as exact values whenever suitable.*
☒	☐	For Bayesian analysis, information on the choice of priors and Markov chain Monte Carlo settings
☒	☐	For hierarchical and complex designs, identification of the appropriate level for tests and full reporting of outcomes
☐	☒	Estimates of effect sizes (e.g. Cohen's d, Pearson's r), indicating how they were calculated

Our web collection on statistics for biologists contains articles on many of the points above.

Software and code

Policy information about availability of computer code

Data collection	TCGA data was collected from cBioportal or UCSC-Xena.
Data analysis	Analysis of the EpiTOF data was performed using an R based pipeline described in Nowicka et al, 2017.<br>UMAPs were generated using the CATALYST package (version 1.12.2).<br>Split violin plots were generated using Vioplot (v0.3.7) R package<br>FACS analysis was performed using FlowJo software v10.2 (Tree Star) or GuavaSoft (v3.1.1)<br>ATAC-seq open regions were detected using MACS2 (v2.0.10.20131216).<br>RNA-seq normalization of counts and differential expression analysis was performed using DESeq2.<br>Normalized log2(norm_count+1) expression was extracted from the TCGA-BRCA dataset and depicted using XENA webtool.<br>Western blots were imaged using ImageLab (v4.1).

For manuscripts utilizing custom algorithms or software that are central to the research but not yet described in published literature, software must be made available to editors and reviewers. We strongly encourage code deposition in a community repository (e.g. GitHub). See the Nature Portfolio guidelines for submitting code & software for further information.

Data

Policy information about availability of data

All manuscripts must include a data availability statement. This statement should provide the following information, where applicable:

- Accession codes, unique identifiers, or web links for publicly available datasets
- A description of any restrictions on data availability
- For clinical datasets or third party data, please ensure that the statement adheres to our policy

The RNA-Seq and ATAC-Seq data generated in this study were aligned to mouse genome assembly (mm10) and have been deposited in NCBI's Gene Expression Omnibus database and are accessible through GEO Series accession number GSE195716.
The CyTOF data was uploaded to the FlowRepository, FR-FCM-Z5L5 and FR-FCM-Z5L6.
All other relevant data are available from the corresponding authors upon reasonable request.

Field-specific reporting

Please select the one below that is the best fit for your research. If you are not sure, read the appropriate sections before making your selection.

☒ Life sciences ☐ Behavioural & social sciences ☐ Ecological, evolutionary & environmental sciences

For a reference copy of the document with all sections, see nature.com/documents/nr-reporting-summary-flat.pdf

Life sciences study design

All studies must disclose on these points even when the disclosure is negative.

Sample size	No sample size calculations were performed. Animal sample size was determined by the literature and the number of biological replicates necessary for ensuring statistical significance. 5 WT-PyMT mouse tumors were used for in vivo studies. 3 PyMT cell lines for each genotype were independently derived. The number of biological replicates are reported in the relevant figure legends in the manuscript.
Data exclusions	No data was excluded.
Replication	Experiments were performed at least 2 times independently. Similar observation was obtained for each replicate and replicated data points are presented in the figures.
Randomization	Not relevant for our study. Comparisons were performed between samples with determined status.
Blinding	The experiments and analyses were performed in a non-blinded fashion since the same investigators performed group allocations during data collection and analysis. Our analyses all generate objective outcomes that are not subject to observer bias.

Reporting for specific materials, systems and methods

We require information from authors about some types of materials, experimental systems and methods used in many studies. Here, indicate whether each material, system or method listed is relevant to your study. If you are not sure if a list item applies to your research, read the appropriate section before selecting a response.

Materials & experimental systems

n/a	Involved in the study	
☐	☒	Antibodies
☐	☒	Eukaryotic cell lines
☒	☐	Palaeontology and archaeology
☐	☒	Animals and other organisms
☐	☒	Human research participants
☒	☐	Clinical data
☒	☐	Dual use research of concern

Methods

n/a	Involved in the study	
☒	☐	ChIP-seq
☐	☒	Flow cytometry
☒	☐	MRI-based neuroimaging

Antibodies

Antibodies used	All antibodies are commercially available:<br>EpiTOF antibodies<br>EpCAM Anti- Mouse CD326 [EpCAM] (G8.8)-166Er 3166014 FLUIDIGM<br>CD49f Anti-Human/Mouse CD49F (GoH3)-164Dy—100 Tests 3164006B FLUIDIGM<br>CD45 Anti-Mouse CD45 (30-F11)-89Y—100 Tests 3089005B FLUIDIGM<br>CD24 Anti-Mouse CD24 150Nd (cancer stem cell marker) 3150009B FLUIDIGM

CD44 Anti-Human/ Mouse CD44 171Yb (cancer stem cell marker) 3171003 FLUIDIGM
a-SMA alpha SMA (Smooth muscle actin) (fibroblast marker) ab5694 Abcam
GATA3 Anti-Human/ Mouse GATA3 167Er 3167007A FLUIDIGM
ERa Anti-Estrogen Receptor alpha antibody [E115] - Low endotoxin, Azide free ab167611 Abcam
beta-Catenin Anti-Human/ Mouse/ Rat beta-Catenin 147Sm 3147005 FLUIDIGM
CyclinB Anti-Human/ Mouse CyclinB1 3153009A FLUIDIGM
Ki-67 Anti- Human Ki-67 (B56) 162Dy 3162012 FLUIDIGM
p53 Anti-Human p53 3143018 FLUIDIGM
OCT3/4 Anti-Human/Mouse Oct3/4 (40/Oct-3)-165Ho 50 Tests 3165023A FLUIDIGM
AREG Anti-Human/ Mouse AREG LS-C341363 LSBio
p21 Anti-p21 antibody [EPR18021] - BSA and Azide free ab232512 Abcam
ZEB1 EMT azide and BSA free NBP2-81015 Novus
YAP D8H1X CST-14074-BF CST
EZH2 D2C9 CST-5246-BF CST
LATS1 (C66B5) CST-3477-BF CST
pYAP (S127) D9W2I CST-13008 CST
H3 Anti-Histone 3 (D1H2)-176Yb 50 Tests 3176016A FLUIDIGM
pH3 Anti-Human/ Mouse/ Rat pHistone H3 [Ser28] 3175012A FLUIDIGM
cleaved H3 Rabbit monoclonal anti-cleaved-Histone H3 (Thr22) (clone D7J2K) CST-12576 (custom formulation*) CST
H3K27ac Acetyl-Histone H3 (Lys27) (D5E4) XP® Rabbit mAb #8173 CST-8173P CST
H3K36me3 Tri-Methyl-Histone H3 (Lys36) (D5A7) XP® Rabbit mAb #4909 CST-4909 CST
H3.3 Rabbit monoclonal anti-Histone H3.3 (clone EPR17899) ab176840 Abcam
H3K4me3 Tri-Methyl-Histone H3 (Lys4) (C42D8) Rabbit mAb #9751 CST-9751 CST
H3K27me3 Mouse monoclonal anti-trimethyl-Histone H3 (Lys27) (clone MABI 0323) Active Motif-61017 Active Motif
H3K4me1 Mono-Methyl-Histone H3 (K4) (D1A9) XP(R) Rabbit mAb, 100 ul CST-5326S CST
H3K9me3 Tri-Methyl-Histone H3 (Lys9) (D4W1U) Rabbit mAb #13969 CST-13969S CST
H2B Recombinant Anti-Histone H2B antibody [EP957Y] - BSA and Azide free ab239842 Abcam
H4K16Ac Acetyl-Histone H4 (Lys16) (E2B8W) Rabbit mAb CST-13534 CST
H3K36me2 Di-Methyl-Histone H3 (Lys36) (C75H12) Rabbit mAb #2901 CST-2901 CST
H3K64ac Anti-Histone H3 (acetyl K64) antibody [EPR20713] - BSAand Azide free, 100 ug ab251549 Abcam

other antibodies
LATS1 (C66B5) Rabbit mAb CST-3477 CST
LATS1 LATS1 antibody HPA031804 Sigma
GAPDH (14C10) Rabbit mAb CST-2118 CST
MYC-tag 9E10 ab32 Abcam
H3 Anti-Histone H3 antibody - Nuclear Marker and ChIP Grade (ab1791) ab1791 Abcam
H3K27ac Acetyl-Histone H3 (Lys27) (D5E4) XP® Rabbit mAb CST-8173P CST
H3K36me2 Di-Methyl-Histone H3 (Lys36) (C75H12) Rabbit mAb (Alexa Fluor® 647 Conjugate) CST-15090 CST
ERa Recombinant Anti-Estrogen Receptor alpha antibody [E115] - ChIP Grade (ab32063) ab32063 Abcam
EpCAM-APC CD326 (EpCAM), mouse 130-102-234 Miltenyl
EpCAM-APC anti human CD326 (Ep-CAM) 9C4 324208 Biolegend
CD49f-PE human & mouse 130-119-767 Miltenyl
YAP/TAZ YAP/TAZ (D24E4) Rabbit mAb CST-8418 CST
GFP Anti-GFP from mouse IgG1κ (clones 7.1 and 13.1) 11-814-460001 Roche
NCOR1 NCoR1 Antibody CST-5948 CST
NCOR1 NCoR1 Antibody ab3482 Abcam
NCOR1 NCoR1 Antibody (F-1) sc-515934 Santa Cruz
HDAC1 HDAC1 (D5C6U) XP® Rabbit mAb CST-34589 CST
KRT8 Anti-Cytokeratin 8 Antibody, clone TROMA-1 MABT329 Sigma
KRT14 Anti-Cytokeratin 14 Antibody (LL001) sc-53253 Santa Cruz
b-ACTIN beta Actin Antibody monoclonal mouse nonconjugated A00702 GenScript
GaR-488 Alexa Fluor 488 dyed secondary antibody A32731 ThermoFischer
GaR-647 Goat anti-Rabbit IgG Cross-Adsorbed Secondary Antibody, Alexa Fluor™ 647 A21244 ThermoFischer
GaM-594 Goat anti-Mouse IgG Cross-Adsorbed Secondary Antibody, Alexa Fluor™ 594 A11032 ThermoFischer

Validation

All antibodies were validated by their respective manufacturer. The LATS1 antibody (CST, #3477) was validated by CST https://www.cellsignal.com/products/primary-antibodies/lats1-c66b5-rabbit-mab/3477?site-search-type=Products&N=4294956287&Ntt=lats1&fromPage=plp and also validated for mouse LATS1 by us by Western blot comparison of WT LATS1 vs LATS1-null lysates (see figure 4b).

Eukaryotic cell lines

Policy information about cell lines

Cell line source(s)

MDA-MB-468, MCF 10A and HEK293T-phoenix cells were obtained from ATCC. PyMT-derived cell lines were generated in house (as described in Methods section of manuscript).

Authentication

none of the cell lines were authenticated.

Mycoplasma contamination

All cell lines tested negative for mycoplasma.

Commonly misidentified lines
(See ICLAC register)

No commonly misidentified cell lines were used in this study.

Animals and other organisms

Policy information about studies involving animals; ARRIVE guidelines recommended for reporting animal research

Laboratory animals	Mice were housed in a pathogen-free facility in single-unit cages with 12-hr alternate light and dark cycles and at controlled ambient temperature (21-23 C) with humidity between 40%-60% with free access to water and irradiated food.<br>For PyMT tumor samples, tumors were harvested from five individual 3.5 month old FBV/N-PyMT female mice (purchased from Jackson, strain #002374). Maximum tumor size (10% of body weight) permitted by the Animal Ethics Committee was not exceeded.
Wild animals	This study did not involve wild animals.
Field-collected samples	This study did not involve samples collected from the field.
Ethics oversight	All mouse experiments were approved by the Institutional Animal Care and Use Committee (IACUC) of the Weizmann Institute (approval #06320720-2).

Note that full information on the approval of the study protocol must also be provided in the manuscript.

Human research participants

Policy information about studies involving human research participants

Population characteristics	The breast cancer sub-study (N=16) derive from a German prospective observational trial of ER-positive, postmenopausal female patients with early-stage invasive breast cancer diagnosis, who were recruited between 2005 and 2011 (N=1286). The median age at diagnosis of the N=16 samples is 63 years (range 51-75 years). Treatment included surgical removal, followed by radiation (N=10), chemotherapy (N=4), and intended 5 years of endocrine treatment (Tamoxifen N=5, Aromatase inhibitors N=7, agent switch usually after 2.5 years N=4).
Recruitment	The study is multicentric with >25 national hospitals plus one centre from Liverpool, UK, for patients recruitment. Patients represent incidental cases from the respective hospital. There is no evidence for selection bias.
Ethics oversight	The study was carried out in accordance with the provisions of the declaration of Helsinki of 1975 and ethics approval was obtained from the Ethics Commission of the University of Tübingen, Germany, and respective local ethics committees of participating centers.<br>The immunohistological assays of breast cancer patient samples presented in this study complied to the Weizmann Institutional Review Board (IRB) approval.

Note that full information on the approval of the study protocol must also be provided in the manuscript.

Flow Cytometry

Plots

Confirm that:

- [x] The axis labels state the marker and fluorochrome used (e.g. CD4-FITC).
- [x] The axis scales are clearly visible. Include numbers along axes only for bottom left plot of group (a 'group' is an analysis of identical markers).
- [ ] All plots are contour plots with outliers or pseudocolor plots.
- [x] A numerical value for number of cells or percentage (with statistics) is provided.

Methodology

Sample preparation	Single cell suspensions were incubated with EpCAM-APC (Miltenyi #130-102-234, 1:100) and CD49f-PE (Miltenyi #130-119-767, 1:500) for 10min in dark at 4C. Unstained samples and incubation with each antibody separately served as controls. Cells were washed and resuspended in FACS buffer (0.5% BSA and 2mM EDTA in PBS) and immediately analyzed.
Instrument	For FACS analyses a Guava EasyCyte (Milipore) was used. For FACS-based sorting a FACSAria III instrument (BD Biosciences) was used.
Software	FlowJo software v10.2 (Tree Star) and GuavaSoft (v3.1.1).
Cell population abundance	FACS-based separation populations generated 1x10^6 WT luminal cells, 148,000 WT basal-like cells, 1x10^6 L1 luminal cells and 920,000 L1 basal-like cells.<br>In a replicate experiment, 1x10^6 WT luminal cells, 150,000 WT basal-like cells, 0.5x10^6 L1 luminal cells and 1x10^6 L1 basal-like cells.

Gating strategy

SSC-A vs FSC-A were used to gate out debris. From this gate, FSC-W vs FSC-H were used to gate out doublets. From this gate, for in vivo DAPI-stained samples, PE-A vs APC-A were used to gate out dead cells. In in vivo and in vitro samples, using EpCAM-APC (Miltenyl #130-102-234, 1:100) and CD49f-PE (Miltenyi #130-119-767, 1:500), unstained and single stained samples were used to generate gates for cell sorting and analysis.

☒ Tick this box to confirm that a figure exemplifying the gating strategy is provided in the Supplementary Information.
